# Controlled Release Strategies for the Combination of Fresh and Lyophilized Platelet-Rich Fibrin on Bone Tissue Regeneration

**DOI:** 10.1155/2019/4923767

**Published:** 2019-05-16

**Authors:** Zhongshuang Liu, Han Jin, Qi Xie, Zhuling Jiang, Shouli Guo, Ying Li, Bin Zhang

**Affiliations:** ^1^Institute of Hard Tissue Development and Regeneration, The Second Affiliated Hospital of Harbin Medical University, Harbin 150001, Heilongjiang, China; ^2^Heilongjiang Academy of Medical Sciences, Harbin 150001, Heilongjiang, China; ^3^Department of Stomatology, Harbin Children's Hospital, Harbin 150001, Heilongjiang, China; ^4^Department of Implantology, The Second Affiliated Hospital of Harbin Medical University, Harbin 150001, Heilongjiang, China; ^5^Animal Experiment Center of the Second Affiliated Hospital, Harbin Medical University, Harbin 150001, Heilongjiang, China

## Abstract

The aim of the present study was to investigate growth factors release kinetics for the combination of fresh platelet-rich fibrin (F-PRF) and lyophilized PRF (L-PRF) with different ratios to promote bone tissue regeneration. First, we quantified the level of transforming growth factor-*β*1 (TGF-*β*1), vascular endothelial growth factor (VEGF), and platelet-derived growth factor-AB (PDGF-AB)* in vitro* and analyzed their release kinetics from F-PRF, L-PRF, and the fresh/lyophilized PRF in different weight ratios (F:L=1:1, 1:3, 1:5). The second experimental phase was to investigate the proliferation and differentiation of bone mesenchymal stem cells (BMSCs) as a functional response to the factors released. To further test the osteogenic potential* in vivo*, different scaffolds (F-PRF, or L-PRF, or F:L=1:1) were implanted in rabbit cranial bone defects. There was a statistically significant increase in proliferation and differentiation of BMSCs when the culture medium contained different PRF exudates collected at day 14 compared with the negative control group. The results showed that the new bone formation in the fresh/lyophilized PRF (1:1) was much more than that of other groups in defects at both 6 and 12 weeks. Our data suggested growth factor concentration and release kinetics as a consequence of fresh and lyophilized PRF combination, which is an effective way for promoting bone regeneration.

## 1. Introduction

It has been known for decades that tissue engineering provides a new way for the restoration of the structure and function of damaged tissue [1, 2]. In recent years, one of the hot topics in tissue engineering is the development of controlled release systems for bone regeneration [3, 4]. However, the complexity of native tissue healing is obviously lacking in current bone regeneration strategies by incorporating the use of single growth factor release [5]. Even if there are a number of design criteria to release kinetics, a single signaling molecule will not satisfy bone regeneration by itself [6]. Thus, the development of multiple growth factors (GFs) release systems for tissue engineering bone has certain enhancement function of biomimetic constructs.

Platelet-rich fibrin (PRF), a second generation of platelet concentrates, has already been widely used in modern medicine [7–9]. In theory, PRF acts as a source of GFs in the early stage of bone repair and regeneration. However, owing to the fact that PRF is an autologous product, limitation is a total duration of the growth factor release, which is too short to improve the bone reconstruction [10]. Lyophilization is a widely used technique to prepare proteins and platelets, which have important significance in clinical application. Lyophilized, platelet-based materials not only have the advantage of better storage stability potential, but also allow newly grown tissue to immediate access to growth factors [11]. Data from past study indicated that lyophilized PRF allowed for sustained release system for growth factors and it was suitable for the bone tissue reconstruction process [12]. However, these studies face many challenges for the design of GFs sustained release systems capable of matching the complexity of native tissue healing.

Lots of factors may affect GFs secretion and make it difficult to provide the appropriate amount of biological signals for tissue engineering [13]. Upon injury, extremes of GFs release were found to be undesirable and controlled and sustained profile should be designed. Therefore, it was expectable to mimic the biological function of native extracellular matrix, which had a significant effect on cell activities and new tissue formation. Although the use of either platelet-rich preparations or L-PRF has been alone tested for skeletal engineering, a combination of both interventions may offer best opportunity for beneficial clinical outcomes. This study aims for a biomimetic strategy that is based on the combination of fresh and lyophilized PRF with different ratios, tailored for different delivery rates of GFs in tissue healing and regeneration. To evaluate this new delivery system, the study was to (1) quantify the level of TGF*β*-1, VEGF, and PDGF-AB* in vitro* and analyze their release kinetics from F-PRF, L-PRF, and PRF and lyophilization with different ratios; (2) investigate the proliferation and differentiation of bone mesenchymal stem cells (BMSCs) as a functional response to the factors released; and (3) evaluate the tissue compatibility and the potential for the reconstruction of the defects of different scaffolds implanted in rabbit cranial defects.

## 2. Materials and Methods

### 2.1. Preparation of Fresh and Lyophilized PRF

Five milliliters of autologous blood in 5 mL coated glass tubes without anticoagulants was obtained from the central ear artery of New Zealand white rabbits. The whole blood was immediately centrifuged at 3000 rpm for 10 min (Labofuge 400Rcentrifuge, Heraeus, Hanau, Germany) according to the PRF protocol [7]. The PRF clots identified as the middle layer were removed from the centrifuge tube and then were gently placed in sterile gauze. For the preparation of lyophilized PRF, PRF clots were frozen and stored at −80°C and then freeze-dried overnight using a Labconco lyophilizer at −51°C (Free Zone, Labconco, Kansas City, MO, USA).

### 2.2. Scanning Electron Microscope (SEM) Analysis

To identify the ultrastructure of the fresh and lyophilized PRF, the PRF derived from two rabbits was randomly selected for observation. Fresh and lyophilized PRF was fixed with a solution of 2.5% glutaraldehyde at 4°C for 1 h and then dehydrated using graded ethanol. Samples were coated with gold and examined with a scanning electron microscope at magnifications of 500 × and 2000 × using an acceleration potential of 10 keV (JSM-5800LV, JEOL, Tokyo, Japan).

### 2.3. Quantification of Growth Factors in the PRF

The PRF clots were gently placed in 5 mL centrifuge tube. The weight of the empty centrifuge tube was recorded as W_0_. After adding fresh PRF, the weight was recorded as W_f+0_. Then the centrifuge tube with fresh PRF was placed in the freeze-dryer and the PRF was completely freeze-dried. The weight of centrifuge tube containing lyophilized PRF was recorded as W_l+0_. Finally, fresh PRF weight was recorded as W_f_ = W_f+0_ - W_0_, and lyophilized PRF weight was recorded as W_l_ = W_l+0_ - W_0_. Water loss rate after freeze-drying was recorded as R = (1-W_l_/W_f_) × 100%. The result showed that the average water loss rate of fresh PRF was 90% after being freeze-dried. Therefore, the amount of growth factors in F-PRF (0.2g) can be considered equivalent to L-PRF (0.02g). The groups were as follows: F-PRF (0.2g), L-PRF (0.02g), fresh/lyophilized PRF (1:1) = 0.1g (F-PRF) +0.01g (L-PRF), fresh/lyophilized PRF (1:3) = 0.05g (F-PRF) +0.015g (L-PRF), and fresh/lyophilized PRF (1:5) = 0.03g (F-PRF) +0.017g (L-PRF). The research quantified the capital growth factors released from each PRF group. TGF-*β*1, VEGF, and PDGF-AB were quantified using an enzyme linked immunosorbent assay (ELISA) kit. Briefly, either fresh or lyophilized PRF clots were placed in a 5 mL centrifuge tube containing 2 mL of Dulbecco's Modified Eagle's Medium (DMEM; Life Technologies, Carlsbad, CA, USA) without fetal bovine serum. The tube was placed in an incubator at 37°C for 28 d. The conditioned medium was collected at the times of 1, 4, 7, 14, 21, and 28 days of culture, and an equal volume of medium was added to the tubes. All collected mediums were stored at -80°C and analyzed at the same time to reduce bias. All ELISA kits were purchased from R&D System (Shanghai, China) and used according to the manufacturer's protocol, and the wavelength for ELISA measurement was 450 nm. All assays were tested in triplicate. The results were inferred as the mean standard deviation and analyzed statistically.

### 2.4. In Vitro Study

#### 2.4.1. Cell Culture

BMSCs were obtained from the bone marrow of male New Zealand white rabbits (0.8–1.2 kg) by whole bone marrow adherent culture methods. Rabbit BMSCs were cultured in low-glucose Dulbecco's Modified Eagle's Medium (LG-DMEM; Life Technologies, Carlsbad, CA, USA), supplemented with 10% fetal bovine serum (FBS, Life Technologies), 100 U/mL penicillin, and 100 *μ*g/mL streptomycin (1% PS, Life Technologies). The conditioned medium was prepared as follows. Briefly, either fresh or lyophilized PRF clots ( F-PRF (0.2 g), L-PRF (0.02 g), and F-PRF and L-PRF in different weight ratios (F:L=1:1, 1:3, 1:5)) were placed in a 5 mL centrifuge tube containing 2 mL of LG-DMEM, respectively. The tube was placed in an incubator at 37°C and the conditioned medium was collected at the time of 14 days. After cell attachment, different ratios of fresh and lyophilized PRF conditioned media were added to different wells. Cells were incubated in a humidified atmosphere at 37°C in 5% CO_2_.

#### 2.4.2. Proliferation Assay

BMSCs were seeded at a density of 3500 cells per well in 96-well plates. The 3-(4,5-Dimethylthiazol-2-yl)-2,5-diphenyltetrazolium bromide (MTT) assay (St. Louis, MO, USA) was performed at different time points (1-7 days). Briefly, 15 *μ*l of MTT stock solution (5 g/L) was added to each well, and the reaction mixture was incubated at 37°C for 4 h; supernatants were removed and replaced by 150 *μ*l of dimethyl sulphoxide (DMSO). Absorbance was measured at 490 nm using a microplate reader (RT-6000; Lei Du Life Science and Technology Co., Shenzhen, People's Republic of China).

#### 2.4.3. Quantification of Mineralization Nodules

To induce BMSCs differentiation, we cultured BMSCs in osteogenic induction media containing 10 nM of dexamethasone, 10 mM of *β*-glycerophosphate, and 100 *μ*M of ascorbic acid (Sigma, Sigma Chemical Co., St. Louis, MO, USA). BMSCs were seeded into 24-well cell culture plates at a concentration of 3 × 10^4^ cells/well and the plates were placed into a CO_2_ incubator for 8 h. After cell attachment, different ratios of fresh and lyophilized PRF conditioned media were added to different wells. After 7 and 14 days of coculture, cells were fixed and stained using Alizarin Red S (Sigma, Sigma Chemical Co., St. Louis, MO, USA) for detecting mineralization. The formation process of mineralization nodes was observed with microscope and the color density of matrix mineralization was measured using the Image-Pro Plus software (Image-Pro Plus 6.0; Media Cybernetics, Rockville, MD, USA).

### 2.5. In Vivo Animal Experiment

#### 2.5.1. Animal Surgical Procedure

All the experimental protocols used for this study were approved by the Animal Experiment Ethics Committee of the Second Affiliated Hospital of Harbin Medical University (SYDW2018-062). Twelve male New Zealand white rabbits between 2.8 and 4 kg were included in this study. Each rabbit was anesthetized with an intramuscular injection of ketamine (10 mg/kg) 30 min prior to the operation. The calvarial region was shaved and the skin was sterilized with 10% povidone-iodine. Four 8 mm diameter defects were created with a trephine bur (3i Implant Innovation, Palm Beach Gardens, FL, USA) with copious irrigation. The four calvarial defects were randomly divided into four groups: F-PRF (0.2g) group, L-PRF (0.02g) group, fresh/lyophilized PRF (1:1, 0.1g (F-PRF) +0.01g (L-PRF)) group, and a control group (non-PRF). Then, the defects were treated with different grafting materials, and subsequently the periosteum, muscle, and skin were sutured. The day of surgery was assigned as day 0. All animals were kept in a single cage and fed a standard dried diet and water.

#### 2.5.2. Radiography and Micro-CT Scanning

The rabbits were sacrificed with an overdose of 200 mg/ml pentobarbital sodium at 6 weeks and 12 weeks after surgery. The entire cranium was extracted with a reciprocating saw and stored in 4% paraformaldehyde. Radiographs were taken of the rabbit cranium by a Faxitron Specimen Radiography System (Model MX-20; Faxitron X-ray Corporation, Wheeling, IL) at 26 kVp and exposure time of 11 s. The degree of bone formation was examined by a micro-CT scanner (*μ*CT35, Scanco Medical AG, Bassersdorf, Switzerland) with a 18.5 *μ*m voxel size using the following parameters: 114 mA, 70 kVp, and exposure time of 300 ms. The new bone formation was calculated as the percentage fraction of new bone area to the total defect area by Image Pro Plus.

#### 2.5.3. Histomorphometric Analysis

After radiography, the calvarial specimens were decalcified in 10% ethylene diamine tetraacetic acid, sectioned by bisecting the 8 mm diameter defects, and then embedded in paraffin. Serial sections in 4 *μ*m were cut from the middle part and stained with hematoxylin and eosin staining (H&E). Histologic evaluation was performed at 10 and 100 magnification using a light microscope (BX50, Olympus Optical, Tokyo, Japan).

### 2.6. Statistical Analysis

Data was analyzed using SPSS 13.0 (SPSS Inc., Chicago, IL, USA). One-way analysis of variance by Tukey's post hoc analysis was used to compare the differences of the mean OD and the percentage of newly formed bone in each group. A* P* value < 0.05 was considered to be significantly different in all cases.

## 3. Results 

### 3.1. Macro- and Microphotographs of Fresh and Lyophilized PRF

**Figure 1:**
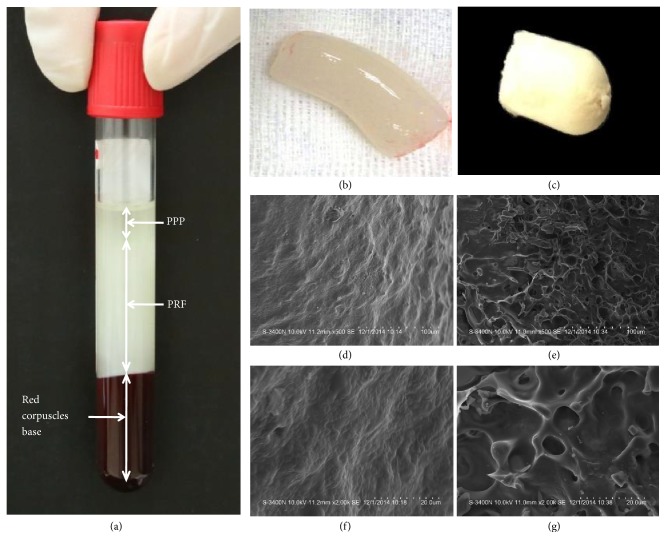
*Macroscope and scanning electron microscope (SEM) analysis of fresh and lyophilized PRF*. (a) Blood centrifugation immediately after collection allowed the composition of a structured and resistant fibrin clot (PRF) to be in the middle of the tube, just between the red corpuscles at the bottom and acellular plasma (PPP) at the top. (b,c) were macrophotographs of fresh and lyophilized PRF preparations. (d-g) were SEM at 500-fold magnification (d,e) and 2000-fold magnification (f,g). (b, d, f) were from fresh PRF and (c, e, g) were from lyophilized PRF.

The PRF was present as a fibrin clot in the middle of the tube after centrifugation, just between the red corpuscles at the bottom and acellular plasma at the top (Figure 1(a)). Macrophotographs of fresh and lyophilized PRF preparations were shown in Figures 1(b) and 1(c). SEM revealed that there was a fiber-like appearance of the fresh PRF (Figures 1(d) and 1(f)), while lyophilized PRF resembled a sponge (Figures 1(e) and 1(g)), resulting in a larger pore size in lyophilized versus fresh PRF.

### 3.2. Quantification of Growth Factors Release Kinetics

Differential growth factor concentration and dynamics of release were observed among the five platelet preparations throughout the experimental period (Table 1). Various TGF-*β*1 release patterns were measured in different groups. F-PRF and fresh/lyophilized PRF (1:3) released the maximum level of TGF-*β*1 at day 7. However, levels of TGF-*β*1 had a peak release at day 14 for L-PRF, fresh/lyophilized PRF (1:1), and fresh/lyophilized PRF (1:5). At day 21, TGF-*β*1 levels of fresh/lyophilized PRF (1:1, 376.75 ± 54.26) were statistically higher than those in fresh/lyophilized PRF (1:3, 240.99 ± 81.25*, P *= 0.015 < 0.05) and fresh/lyophilized PRF (1:5, 264.81 ± 75.64,* P *= 0.022 < 0.05). VEGF levels of fresh/lyophilized PRF (1:1, 64.62 ± 6.29) at day 4 were statistically higher than those of L-PRF (46.05 ± 8.88,* P *= 0.020 < 0.05) and fresh/lyophilized PRF (1:5, 41.35 ± 1.73, P = 0.005 < 0.01). At day 7, fresh/lyophilized PRF (1:1, 108.08 ± 19.79) showed the higher concentration when compared with fresh/lyophilized PRF (1:3, 72.49 ± 3.85), which was statistically highly significant (*P *= 0.044 < 0.05). However, at the same time points, no statistically significant difference in levels of PDGF-AB could be identified among the groups.

### 3.3. Effect of Fresh/Lyophilized PRF on BMSCs Proliferation and Differentiation

**Figure 2:**
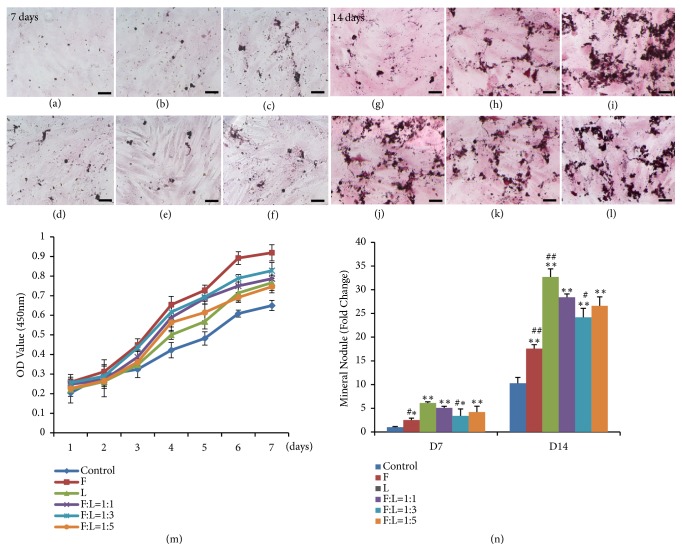
*Effects of fresh and lyophilized PRF on BMSCs proliferation and mineralization*. The five different conditional media used in the proliferation and mineralization study, fresh PRF, lyophilized PRF, and the fresh/lyophilized PRF conditional media at different concentrations (F:L=1:1, 1:3, 1:5) and with DMEM medium as a control. In (a-f, g-l), alizarin red staining in BMSCs cultured for 7 and 14 days was compared. (a and g) were control group; (b and h) were fresh PRF group; (c and i) were lyophilized PRF group; (d and j) were F:L=1:1 group; (e and k) were F:L=1:3 group; (f and l) were F:L=1:5 group. (m) illustrated the results of BMSCs proliferation assays and (n) were based on results from alizarin red S mineralization assays. Scale bars represent 100 *μ*m. Statistically significant difference compared with control group *∗∗P* < 0.01, *∗P* < 0.05, with fresh/lyophilized PRF (1:1) group, ^**##**^*P* < 0.01,^** #**^*P* < 0.05.

As for the MTT assay, the absorbance values for the growth curves for all of the groups increased during the testing period (Figure 2(m)). There was a statistically significant increase in proliferation of BMSCs when the culture medium contained different PRF exudates collected at day 14 compared with the negative control group (*P *< 0.05).

Following 7 days and 14 days, both fresh and lyophilized PRF demonstrated osteoinductive properties and increased mineral nodule formation (Figures 2(a)–2(l)). When cultured for 7 days, cells treated with different PRF conditional media reached relatively higher mineralization than those of the control group (*P* < 0.01). Moreover, compared with the fresh/lyophilized PRF (1:1, 5.07% ± 0.34%) group, there were statistical differences of mineral nodule formation for F-PRF (2.52% ±  0.42%,* P *= 0.042 < 0.05) and fresh/lyophilized PRF (1:3, 3.39% ± 1.45%,* P *= 0.037 < 0.05) groups. After 14 days of culture, the number of nodules obtained in differentiation conditions using different PRF conditional media was much higher than that of control group (*P* < 0.01). Fresh/lyophilized PRF (1:1, 28.4% ±  0.7%) strongly enhanced the osteogenic capacity of BMSCs and increased mineral nodule formation compared to the F-PRF (17.6% ± 0.8%,* P *= 0.007 < 0.01) and fresh/lyophilized PRF (1:3, 24.2% ± 1.9%,* P *= 0.038 < 0.05) group at 14 days (Figure 2(n)).

### 3.4. Effect of Fresh/Lyophilized PRF on Cranial Bone Regeneration

**Figure 3:**
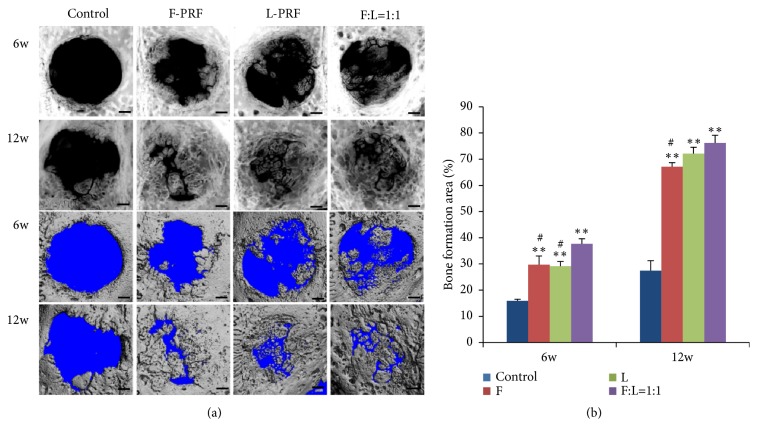
*Bone regeneration in rabbit critical size calvarial defects and quantification after 6 and 12 weeks after operation: radiographs and micro-CT analysis*. 8 mm diameter defects were created and the defects were treated by filling with fresh PRF, lyophilized PRF, or fresh/lyophilized PRF (1:1) or were left unfilled as empty defect controls. (a) Representative radiographs images and micro-CT images. (b) Analysis of the regenerated tissue covering the calvarial defect. Statistically significant difference compared with control group *∗∗P* < 0.001, with fresh/lyophilized PRF (1:1) group, ^#^*P* < 0.01.

To investigate the application of fresh/lyophilized PRF in bone regeneration, critical size cranial bone defects were created and defects were covered with either F-PRF, L-PRF, or fresh/lyophilized PRF (1:1), with the empty defects as a control.  Figure 3 presented the radiographs of calvarial bone that elucidate the osteoconductive potential of F-PRF, L-PRF, or fresh/lyophilized PRF in forming new bones at 6 and 12 weeks. Six weeks after surgery, the empty defects showed homogeneous radiolucent areas over nearly the entire defect. The empty defects treated with F-PRF showed the newly formed bone with irregularly shaped and varying densities, particularly along the defect edge. The empty defects treated with L-PRF showed the same pattern but less radiopacity than the fresh PRF group. Fresh/lyophilized PRF (1:1) showed bone-like density at the margins of the defects and a homogeneous radiolucent area in the central part of the defect. Twelve weeks after surgery, the amounts of radiopaque materials in the PRF construct exceeded those in the radiograph obtained after 6 weeks. This newly formed bone was deposited from the edge of the calvarial bone defect, in the centripetal direction, obscuring the original margin of the calvarial bone defect (Figure 3(a)).

**Figure 4:**
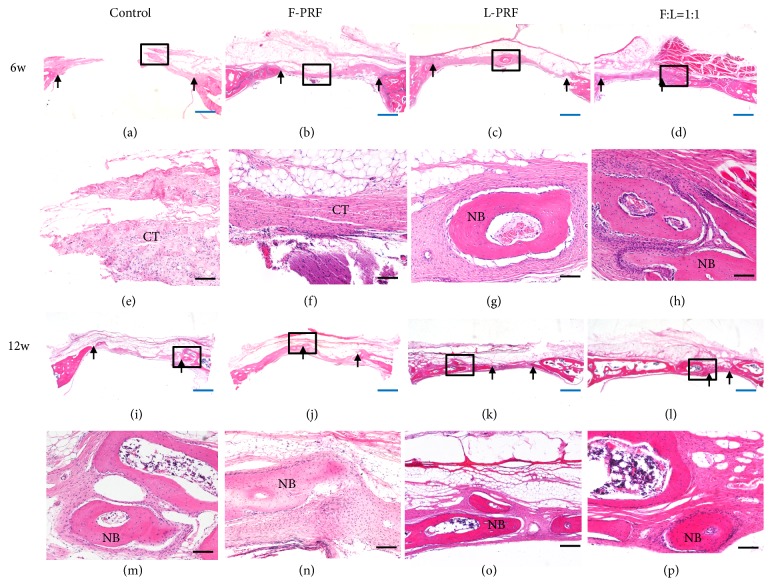
*Hematoxylin and eosin staining of rat cranial defect after 6 or 12 weeks after operation*. Representative light microscopic images of cranial tissue sections of the four groups: controls, fresh PRF, lyophilized PRF, and fresh/lyophilized PRF (F:L=1:1). Black arrows indicate the edge of the defect. H&E staining, (a)-(d), (i)-(l), × 10 magnification, scale bar 1mm (blue); (e)-(h), (m)-(p), × 100 magnification, scale bar 100 *μ*m (black). NB, new bone, CT, connective tissue.

Three-dimensional CT scans of the cranium also revealed an apparently open defect in the control group, while the defect area was reduced in F-PRF and L-PRF groups and the defect nearly closed in fresh/lyophilized PRF (1:1) group after 6 and 12 weeks. Bone formation area was presented in Figure 3(b), which showed that there was significantly greater healing in the PRF groups compared with control group (*P* < 0.001) at 6 weeks. The bone formation area of the fresh/lyophilized PRF (1:1) group was 37.7% ± 1.9%, which was significantly higher than that of the control group (15.9% ± 0.5%,* P* < 0.001), F-PRF (29.7% ± 3.3%,* P* =0.008 < 0.01), and L-PRF group (29.1% ± 1.8%,* P* = 0.005 < 0.01). At 12 weeks, the bone formation areas of different PRF groups were much higher than those of control group (*P* < 0.001), and the fresh/lyophilized PRF (1:1) group had further increased to 77.2% ± 3.0%, which was significantly higher than that of the F-PRF group (65.1% ± 1.6%,* P=*0.004 < 0.01). However, at this time point, no statistically significant difference was observed when comparing fresh/lyophilized PRF (1:1) and L-PRF (71.9% ± 2.4%) group. H and E staining also showed that the new bone formation in the fresh/lyophilized PRF (1:1) group was much more than that of other groups in the original defect margin at both 6 weeks and 12 weeks (Figure 4).

## 4. Discussion

The architecture of the fibrin network is a detrimental feature for the biological properties of the final PRF product. The release of growth factors in PRF serves as the biological basis for tissue healing and regeneration. And previous studies have shown that many kinds of growth factors were released after platelet activation and may control cell behavior and be used to help bone grafts integrate with surrounding bone tissue [14, 15]. Our present study has demonstrated the biomimetic strategy of designed scaffolds for the combination of PRF and lyophilization with different ratios for tissue regeneration.

PRF is a strictly autologous fibrin matrix containing a large quantity of platelet and leukocyte cytokines. Growth factors are released after activation from the platelets trapped within fibrin matrix [7, 8]. Platelets are anucleate cytoplasmic fragments containing *α*-granules. Fibrin is the activated form of a plasmatic molecule called fibrinogen. This soluble fibrillary molecule is massively present in the platelet *α*-granules and plays a determining role in platelet aggregation. The structural integrity of platelets represents an incomplete activated state, which will be transformed into the activated state, accompanied by the continuous release of growth factors after the fibrinolysis [10]. After lyophilization treatment, irregular platelets in shape or incomplete ones in membrane and the decrease of *α*-granule were discovered in lyophilized PRF, which was attributed to the freeze thawing of platelet and the rupture of *α*-granules in the thawing process. This process maybe leads to the fact that the partial growth factors are released to fibrin network and then combine with it. Moreover, the distinctively close association between platelet and fibrin can lead to an effective biological combination which makes a big difference to the process of growth factors release [12, 14]. In fresh PRF, it was observed that each clump of platelet was associated with several fibrins and fibrins stretching along with them. When observed by SEM, lyophilized PRF showed a loose spongy structure with a large number of internal spaces, while there was a fiber-like appearance of the fresh PRF. Obviously, the process of lyophilization could make a big difference to the structure and biological activity of fibrin and platelet. Our study also showed that the release amount and release time of growth factors in F-PRF and L-PRF are different. The kinetics of growth factors release strongly influences tissue regeneration. Most recently, studies have focused on the design and tailoring of appropriate combinations of bioactive factors to match the desired goals regarding tissue regeneration. Therefore, this study tried to combine fresh and lyophilized PRF with different ratios in order to seek delivery systems of bioactive factors for tissue healing.

We studied the secretion profile of three cytokines (TGF-*β*1, VEGF, and PDGF-AB), which are of crucial importance in bone healing. The dynamics of release demonstrated more TGF-*β*1 sustained release from fresh/lyophilized PRF (1:1) group, which had a peak release at day 14, in contrast to released peak value of the growth factor at the first 7 days of other groups. More significantly, fresh/lyophilized PRF (1:1) still retained statistically higher levels of TGF-*β*1 than other groups at day 21, which may lead to maximum mineralization. The knowledge that PRF releases high amounts of TGF-*β*1 and allows sustained release of other growth factors could provide important guidelines for the choice of tissue growth and would repair in future clinical studies [16, 17]. VEGF releases showed similar profile in different groups, characterized by a quick increase of the release during the first 24 h. The fresh/lyophilized PRF (1:1) reached the maximum level of VEGF at day 7. Marx et al. reported that the autologous growth factors have direct effects on cells for 5-7 days. VEGF level of guided bone regeneration was higher than that of the nonbone guided bone regeneration, and the concentration at the first week after the operation is the highest [18]. VEGF is the basis of angiogenesis and bone regeneration which has a promoting effect on guided bone regeneration [19]. PDGF was originally discovered from platelets and released from its *α*-granules during the early stage of damage to initiate the fission and proliferation of osteoblast in the wound [20]. The present* in vitro* study had shown that no statistically significant differences in levels of PDGF were observed among the groups. Several factors can influence the total release as well as the dynamics of growth factors released from platelet concentrates. It was demonstrated in the present study that PRF experienced controllable and long-term release of growth factors. The statistical analysis of levels of TGF-*β*1, VEGF, and PDGF-AB released from fresh/lyophilized PRF at different time points confirmed the former hypothesis that fresh/lyophilized PRF not only prolonged the release of growth factors but also delayed the peak of releasing.

In the same way, we investigated the proliferation and differentiation of BMSCs as a functional response to the factors released. In our study, the addition of F-PRF and L-PRF in the primary cultures of BMSCs in standard conditions seemed to stimulate simultaneously, in a dose-dependent way, the proliferation and some kind of differentiation characterised by the formation of mineralisation nodules. The BMSCs which received L-PRF showed differentiation characteristics which were highly superior to all the other groups. However, no significant differences were observed in proliferation among the cells treated with the lyophilized/fresh PRF conditional media at different concentrations. It was reported that bone graft healing process involved inflammation revascularization, osteogenesis, and bone remodeling, and the proliferation and differentiation of osteoblasts occurred during the initial 14 days [21]. In our study, we investigated the proliferation and the differentiation of BMSCs using the medium collected at day 14, as it is known that the cells react to the growth factors by proliferating or differentiation. The effects of the two key platelet cytokines, TGF-*β*1 and PDGF-AB, are very variable according to the initial state of the cells: the TGF-*β*1 acting rather on the differentiation process and the PDGF-AB on the proliferation process. The effect of growth factor content and release on the BMSCs primary cultures may explain some of our results.

In this study, three-dimensional CT scans of the cranium revealed an apparently open defect in the control group, while the defect area was reduced in fresh or lyophilized PRF groups and the defect nearly closed in fresh/lyophilized PRF group at 12 weeks. These results indicated that lyophilization does exert a positive impact on the PRF to promote bone regeneration, demonstrating that various cytokines and fibrin networks in PRF still keep the capacity to promote the chemotaxis and proliferation of surrounding osteoblasts. In general, these results were consistent with the former one obtained by the histology examination.

## 5. Conclusions

This study aims for a biomimetic strategy that is based on the combination of fresh and lyophilized PRF with different ratios. The results demonstrated that combination of fresh and lyophilized PRF can successfully stimulate osteogenic differentiation of BMSCs* in vitro* and increase bone formation at the bone defect part* in vivo*. Although this study seeks delivery systems of bioactive factors for tissue healing, future studies will determine whether to mimic the complexity of the native ECM by platelet-rich preparations to promote tissue regeneration.

## Figures and Tables

**Table 1:** The levels of growth factors released from PRF (0.2 g), lyophilized PRF (0.02 g), and PRF and lyophilized PRF in different weight ratios (F:L=1:1, 1:3, 1:5) at 1, 4, 7, 14, 21, and 28 days as determined by ELISA.

Group	Amounts of released molecules (mean and standard deviations)					
	D1	D4	D7	D14	D21	D28
TGF-*β*1(ng/l)						
F	275.92±25.02	326.80±89.32	441.98±65.98	329.11±48.64	288.63±86.32	195.75±86.59
L	242.10±95.20	305.34±45.03	393.45±72.14^*∗*^	407.69±115.23	298.16±65.96	279.11±75.36
F:L=1:1	302.98±72.30	293.39±42.13	321.99±75.21^*∗*^	483.92±87.25	376.75±54.26	231.46±68.59
F:L=1:3	321.99±89.25^*∗*^	307.69±84.26	383.45±56.32^*∗*^	312.46±76.58^#^	240.99±81.25^#^	198.11±65.31
F:L=1:5	350.57±49.25	264.82±45.32^*∗∗*^	364.87±75.23^*∗*^	398.82±86.32	264.81±75.64^#^	217.17±78.36

VEGF(ng/l)						
F	67.70±11.97	52.01±6.95	75.40±10.07	44.23±10.75	27.15±6.40	24.37±8.46
L	71.32±15.54	46.05±8.88^#^	79.63±16.78	64.72±5.38	23.66±11.68	24.19±5.90
F:L=1:1	65.34±6.61	64.62±6.29	108.08±19.79	65.89±22.66	24.04±1.34	23.56±8.78
F:L=1:3	62.27±1.96	51.10±1.52	72.49±3.85^#^	73.85±4.27	21.73±6.21	18.27±2.51
F:L=1:5	61.91±6.71	41.35±1.73^##^	78.10±6.80	54.23±7.11	27.33±5.25	17.10±2.33

PDGF-AB(ng/l)						
F	409.15±61.90	515.27±62.91	402.25±99.95	426.29±8.21	263.01±51.09	142.05±29.53
L	468.51±79.09	454.97±49.66	525.74±35.58	396.91±79.65	312.18±40.66	165.41±17.79
F:L=1:1	398.87±57.84	487.07±59.87	508.65±55.23	420.23±111.51	292.32±51.17	127.35±24.75
F:L=1:3	439.38±35.69	530.56±11.78	450.35±17.59	458.11±22.36	289.10±12.19	170.27±21.59
F:L=1:5	423.71±95.54	432.64±40.71	424.86±81.40	463.30±28.30	369.92±41.32	119.90±16.28

Abbreviations: TGF-*β*1, transforming growth factor-*β*1; VEGF, vascular endothelial growth factor; PDGF-AB, platelet-derived growth factor. Statistically significant difference compared with F-PRF group, *∗∗P* < 0.01, *∗P < 0.05, *with fresh/lyophilized PRF (1:1) group, ^##^*P < 0.01*, ^#^*P < 0.05.*

## Data Availability

The data used to support the findings of this study are available from the corresponding author upon request.
